# Explanation and prediction of clinical data with imbalanced class distribution based on pattern discovery and disentanglement

**DOI:** 10.1186/s12911-020-01356-y

**Published:** 2021-01-09

**Authors:** Pei-Yuan Zhou, Andrew K. C. Wong

**Affiliations:** Systems Design Engineering, University of Waterloo, Waterloo, Ontario Canada

**Keywords:** Pattern discovery, Disentanglement, Clinical decision-making, Imbalance classification

## Abstract

**Background:**

Statistical data analysis, especially the advanced machine learning (ML) methods, have attracted considerable interest in clinical practices. We are looking for interpretability of the diagnostic/prognostic results that will bring confidence to doctors, patients and their relatives in therapeutics and clinical practice. When datasets are imbalanced in diagnostic categories, we notice that the ordinary ML methods might produce results overwhelmed by the majority classes diminishing prediction accuracy. Hence, it needs methods that could produce explicit transparent and interpretable results in decision-making, without sacrificing accuracy, even for data with imbalanced groups.

**Methods:**

In order to interpret the clinical patterns and conduct diagnostic prediction of patients with high accuracy, we develop a novel method, Pattern Discovery and Disentanglement for Clinical Data Analysis (cPDD), which is able to discover patterns (correlated traits/indicants) and use them to classify clinical data even if the class distribution is imbalanced. In the most general setting, a relational dataset is a large table such that each column represents an attribute (trait/indicant), and each row contains a set of attribute values (AVs) of an entity (patient). Compared to the existing pattern discovery approaches, cPDD can discover a small succinct set of statistically significant high-order patterns from clinical data for interpreting and predicting the disease class of the patients even with groups small and rare.

**Results:**

Experiments on synthetic and thoracic clinical dataset showed that cPDD can 1) discover a smaller set of succinct significant patterns compared to other existing pattern discovery methods; 2) allow the users to interpret succinct sets of patterns coming from uncorrelated sources, even the groups are rare/small; and 3) obtain better performance in prediction compared to other interpretable classification approaches.

**Conclusions:**

In conclusion, cPDD discovers fewer patterns with greater comprehensive coverage to improve the interpretability of patterns discovered. Experimental results on synthetic data validated that cPDD discovers all patterns implanted in the data, displays them precisely and succinctly with statistical support for interpretation and prediction, a capability which the traditional ML methods lack. The success of cPDD as a novel interpretable method in solving the imbalanced class problem shows its great potential to clinical data analysis for years to come.

## Background

Clinical diagnostic decisions have a direct impact on the outcomes and treatment of patients in the clinical setting. As large volumes of biomedical and clinical data are being collected and becoming available for analysis, there is an increasing interest and need in applying machine learning (ML) methods to diagnose diseases, predict patient outcomes and propose therapeutic treatments. Today, Deep Learning (DL) has been successful in assisting analysis and classifying medical scans, especially those forms of visual data. However, when dealing with relational datasets where no explicit pattern (except the class label if given) could be extracted from the input data to relate to the decision targets, the ML/DL process remains opaque. In addition, existing ensemble algorithms, such as Boosted SVM, or Random Forest could produce good predictive results, but the underlying patterns in support of the decision are still opaque and uninterpretable for the clinicians [1]. Hence, existing ML approaches on relational data are still encountering difficult problems concerning transparency, low data volume, and imbalance classes [2, 3].

To render transparency and interpretability, Decision Tree, Frequent Pattern Mining or Pattern Discovery were proposed. For decades, *Frequent Pattern Mining* [4–6] is an essential data mining task to discover knowledge in the form of association rules from relational data [6]. The association rules or patterns are made up of co-occurring items or attribute values (AVs) referred to as Attribute Value Associations (AVAs). However, as revealed in our recent work [7–9], the AVA forming patterns of different classes/targets could be overlapping or entangling with each other due to multiple entwining functional characteristics or factors of different groups/classes inherent in the source environments. For example, in the clinical practice, the relation between the input (in terms of inherent patterns apart from the given class label) and the output (decision targets/classes) is not that obvious, particularly when the correlation of signs, symptoms, test results of the patients could be the manifestation of multiple factors. The patterns discovered directly from the acquired data may have overlapping or functionally entwined AVAs as observed from our recent works [7, 9]. We call this pattern entanglement.

**Fig. 1:**
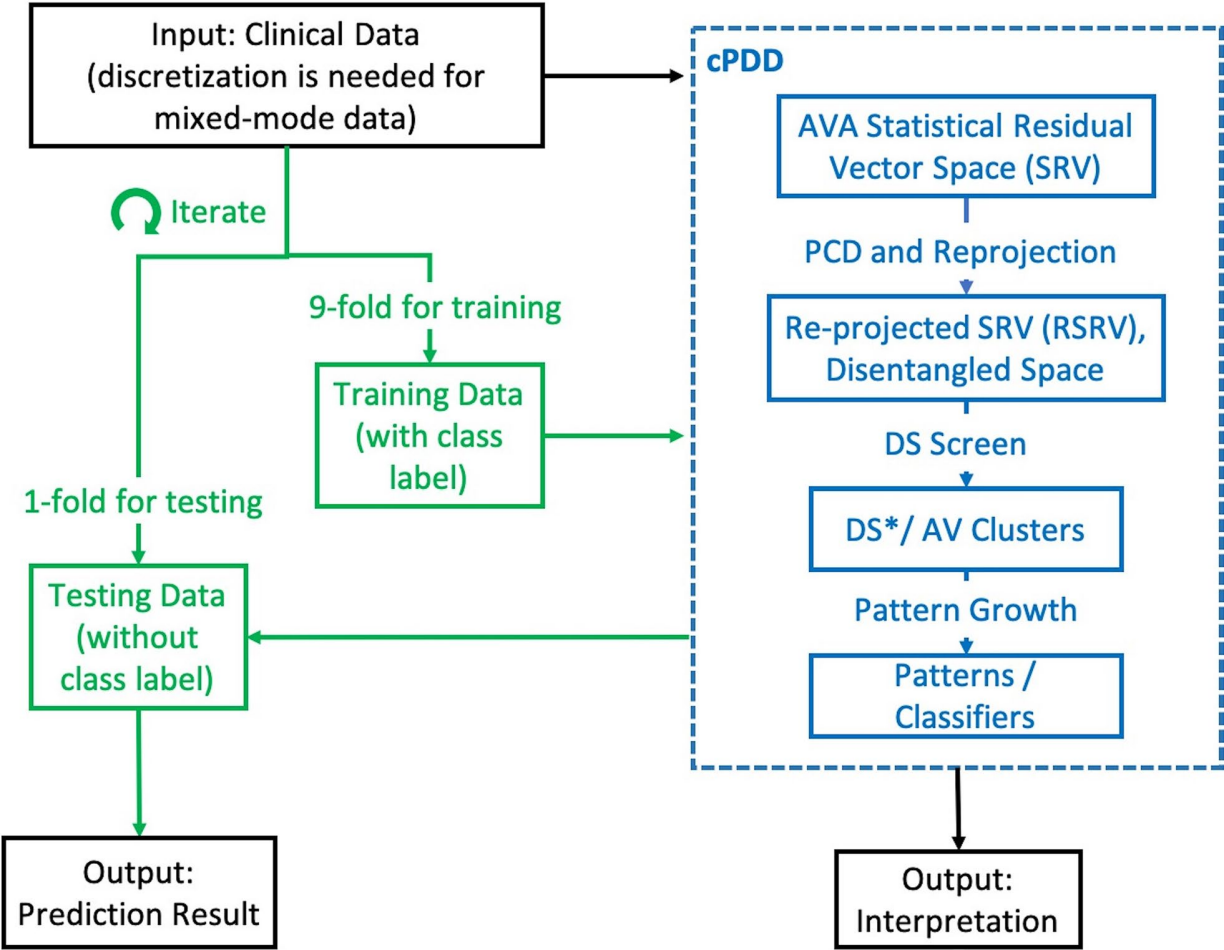
Overview of cPDD for Interpretation and Prediction

Hence, we present a new classification method, called Clinical Pattern Discovery and Disentanglement (cPDD), with novel capability to tackle this problem, particularly focused on the imbalanced class problem. The algorithm is briefly described in Fig. 1 by taking a relational dataset **R** says with *N* attributes as input.

Firstly, Attribute-Value Association Frequency Matrix (AVAFM) is constructed, where the Attribute-Value Association (AVA) is defined as the association between a pair of AVs (from different attributes). The AVAFM consists of the frequency of co-occurrences of all AV pairs within an entity from all entities in **R**. Then, to evaluate the statistical significance of each AVA, the frequency of co-occurrences in AVAFM is converted to a statistical measure known as adjusted statistical residual (SR) [6] accounting the deviation of that frequency from its default model, that is, the frequency of co-occurrences of the AV pairs is statistically independent, i.e., containing no correlated relation. Then, the new matrix is called AVA Statistical Residual Vector Space (SRV) each row of which represents an AV-vector with its coordinates representing the SR values of that AV associated with other AV’s represented by the column vectors. The next step of cPDD is applying principal component decomposition (PCD) to decompose the SRV into different principal components (PCs) and re-project the projections of the AV-vectors on each PC to a new SRV, referred to as Re-projected SRV (RSRV). The AV-vectors with a new set of coordinates in the RSRV reflect the SR of AVAs captured by that PC. The PC and its RSRV together refer to an AVA Disentangled Space (DS). Since the number of DSs is as large as the number of AVs, cPDD only select a small set of DSs denoted by DS* = { ${DS}_i^{\ast }$ } if the maximum SR in the RSRV of that DS exceeds a set statistical threshold (e.g., 1.44 in 85% confidence interval). As the AVs sharing statistically significant AVAs will form Attribute-Value Clusters (AV-Clusters) in a PC reflecting a group of strongly associating AVs. An AV Cluster is defined as a set of AVs such that each of which is associated with an AV of the other attribute in the cluster.

In traditional pattern discovery, to discover high-order patterns from the AVs of a dataset is complex since there is an exponential number of combinations of AVs as pattern candidates. cPDD discovers patterns from each of the small number of AV-Clusters from a small set DS*. Hence, it not only dramatically reduces the number of pattern candidates, but also separates patterns according to their orthogonal AVAs components revealing orthogonal functional characteristic (AVAs) in AV clusters [9, 10] and in subgroups of different DS*. Since the AV-clusters are coming from a disentangled source, the set of patterns discovered therein are relatively small with no or least overlapping and “either-or” cases among their AVs. Thus, cPDD significantly reduces the variance problem and relates patterns to more specific targets/groups. Unlike traditional Pattern Discovery (PD) methods which often produce an overwhelming number of entangled patterns, cPDD renders a much smaller succinct set of patterns associating with specific functionality from the disentangled sources for easy and direct interpretation. Furthermore, due to the reduction of the pattern-to-target variance, the patterns discovered from an uncorrelated AVA source environment will enhance prediction and classification, particularly effectively for data with imbalanced classes.

## Machine learning on clinical data analysis

Today, deep learning (DL) and frequent pattern mining are two commonly used methodologies for data analysis. However, in a more general healthcare setting where data analytics is based predominantly on clinically recorded numeral and descriptive data, the relation between the input (in terms of inherent patterns apart from the given class label) and the output (decision targets/classes) is not that obvious, particularly when the correlation of signs, symptoms, and test results of the patients could be the manifestation of multiple factors [2, 11]. Hence, this poses a challenge to DL in clinical application. Another concern is on the transparency and the assured accuracy [2, 11]. As for transparency, DL is generally considered as a black box [12]. Although ML methods like ensemble algorithms, such as Boosted SVM for imbalanced data (BSI), or Random Forest are good at prediction, their classification results are highly opaque and difficult for the clinicians to interpret [1]. Hence, to render transparency and interpretability, Decision Tree, Frequent Pattern Mining or Pattern Discovery were proposed. Since rules discovered by Decision Tree are guided by class labels, it is unlikely to discover class related AVAs between attributes when class labels are not available. Furthermore, as revealed in our recent work [7–9], AVAs discovered from relational data could be entangled due to multiple entwining functional characteristics inherent in the source environments. The patterns discovered using existing frequent pattern mining approaches based on the likelihood, weight of evidence [6], support, confidence or statistical residuals [5, 6], may have overlapping or functionally entwined AVA patterns inherent in the acquired data leading to overwhelming pattern number and redundancy, making explanation very difficult. Although extra pattern clustering, pruning and summarization algorithms [13, 14] have been proposed and produced a smaller set of patterns/pattern clusters, yet the pattern entanglement problems have not been solved and the interpretation is not comprehensive and succinct.

The cPDD proposed in this paper has solved the fundamental pattern entanglement problem and met the clinical challenges posed above. It intends to provide clinicians with concise and robust clinical patterns discovered from the disentangled sources. The patterns are presented in a more succinct and interpretable form to reveal diagnostic characteristics of the patients and provide statistical support for prediction. Due to its ability of pattern disentanglement, patterns from minority class can be discovered in AVA Statistic Spaces (RSRVs) orthogonal to those of the majority classes.

cPDD extends our recent work [9] on AVA disentanglement to the discovery of statistically significant high-order patterns in AVA disentangled spaces. Its major contributions are three-fold.

i. The cPDD discovers and disentangles statistically significant high-order patterns to reveal the characteristics of different functional subgroups and/or classes in clinical data.
ii. It provides an explicit pattern representation for interpreting the characteristics of the dataset
iii. It uses the discovered patterns to classify entities in the dataset with high precision even when the class distributions are imbalanced.

## Methods

In this section, we extend our previous work, Attribute-Value Association Discovery and Disentanglement Model (AVADD) [9, 10, 15], to cPDD to discover robust and succinct statistically significant high-order patterns and pattern clusters for interpreting and predicting clinical data with imbalanced classes. Table 1 gives an abbreviation of terms and Fig. 1 provides a schematic overview of cPDD.

**Table 1:** Notations and terminologies

AV	Attribute Value
AVA	Attribute Value Association
AV Cluster	Attribute Value Cluster
SR	Adjusted Statistical Residual for an AV pair
SRV	AVA Adjusted Statistical Residual Vector Space
PCD	Principal Component Decomposition
RSRV	Re-projected SRV
DS	Disentangled Space
DS*	Selected Disentangled Space, the selected set

First, we denote the input data as **R**, which contains *N* attributes, denoted as A = { *A*_1_, *A*_2_, …*A*_*N*_ }, and each attribute (*A*_*n*_) is denoted as ${A}_n=\left\{{A}_n^1,{A}_n^2,\dots {A}_n^{I_n}\right\}$, where *I*_*n*_ represents the number of AVs of the nth attribute, *A*_*n*_. The AVA for an AV pair (${A}_n^i$, ${A}_{n\prime}^j$) is represented as ${A}_n^i\leftrightarrow {A}_{n\prime}^j$, which describes the association between “the *ith* value of the *nth* attribute” and “the *jth* value of the *n’th* attribute”. Then, cPDD is implemented in the following five steps.

1. **Statistical Data Analysis:** The measurement that we used here is the same as that used for high-order pattern discovery [6] which uses a statistical method to evaluate the significance of the associations of an AV cluster. From here on, an AVA denotes an association between a pair of AVs (or called AV pair). First, the Frequency Matrix (FM) denoted by a *T* × *T* matrix of AVA relative frequencies between two AVs is constructed, where *T* is the total number distinct AVs in the table. Then the FM is turned into an Adjusted Statistical Residual Vector Space (SRV) to represent the statistical weights of all the AVA pairs obtained from **R**. The dimension of SRV is same as that of FM, and each item of SRV is denoted as a SR $\left({A}_n^i\leftrightarrow {A}_{n\prime}^j\right)$, which represents the adjusted residual between two AVs $\left({A}_n^i\leftrightarrow {A}_{n\prime}^j\right)$. The value of SR $\left({A}_n^i\leftrightarrow {A}_{n\prime}^j\right)$ is calculated by Eq. (1)

1 $$SR\left({A}_n^i\leftrightarrow {A}_{n^{\prime}}^j\right)=r\left({A}_n^i\leftrightarrow {A}_{n^{\prime}}^j\right)v\left({A}_n^i\leftrightarrow {A}_{n\prime}^j\right)$$

where $r\left({A}_n^i\leftrightarrow {A}_{n^{\prime}}^j\right)$ represents the standardized residual of the association.

$$r\left({A}_n^i\leftrightarrow {A}_{n^{\prime}}^j\right)=\frac{Occ\left({A}_n^i\leftrightarrow {A}_{n^{\prime}}^j\right)- Exp\left({A}_n^i\leftrightarrow {A}_{n^{\prime}}^j\right)}{\sqrt{Exp\left({A}_n^i\leftrightarrow {A}_{n^{\prime}}^j\right)}}.$$

$Occ\left({A}_n^i\leftrightarrow {A}_{n^{\prime}}^j\right)$ is the total number of co-occurrences for ${A}_n^i\ and\ {A}_{n^{\prime}}^{j.}$

$Exp\left({A}_n^i\leftrightarrow {A}_{n^{\prime}}^j\right)=\frac{Occ\left({A}_n^i\right) Occ\left({A}_{n^{\prime}}^j\right)}{M}$ is the expected frequency and M is the total number of entities.

$v\left({A}_n^i\leftrightarrow {A}_{n^{\prime}}^j\right)$ represents the maximum likelihood estimate of the variance of $r\left({A}_n^i\leftrightarrow {A}_{n^{\prime}}^j\right)$,

and $$v\left({A}_n^i\leftrightarrow {A}_{n^{\prime}}^j\right)=\mathit{{var}}\left({A}_n^i\leftrightarrow {A}_{n^{\prime}}^j\right)=1-\frac{Occ\left({A}_n^i\right)}{M}\frac{Occ\left({A}_{n^{\prime}}^j\right)}{M}$$

Therefore, SRV is an *T* × *T* matrix representing an adjusted standard residual (SR) Space [6] where *T* represents the total number of distinct AVAs. Hence, SR accounts for the deviation of its observed frequency of occurrences against the expected frequency of occurrences if the AVs in the pair are statistically independent. Generally speaking, the significant associations can be selected according to the threshold obtained from the hypothesis test of statistically significant SR. For example, when the association’s SR > 1.44, it can be treated as positively significant with an 85% confidence level (SR > 1.28 with 80% confidence level).

**Fig. 2:**
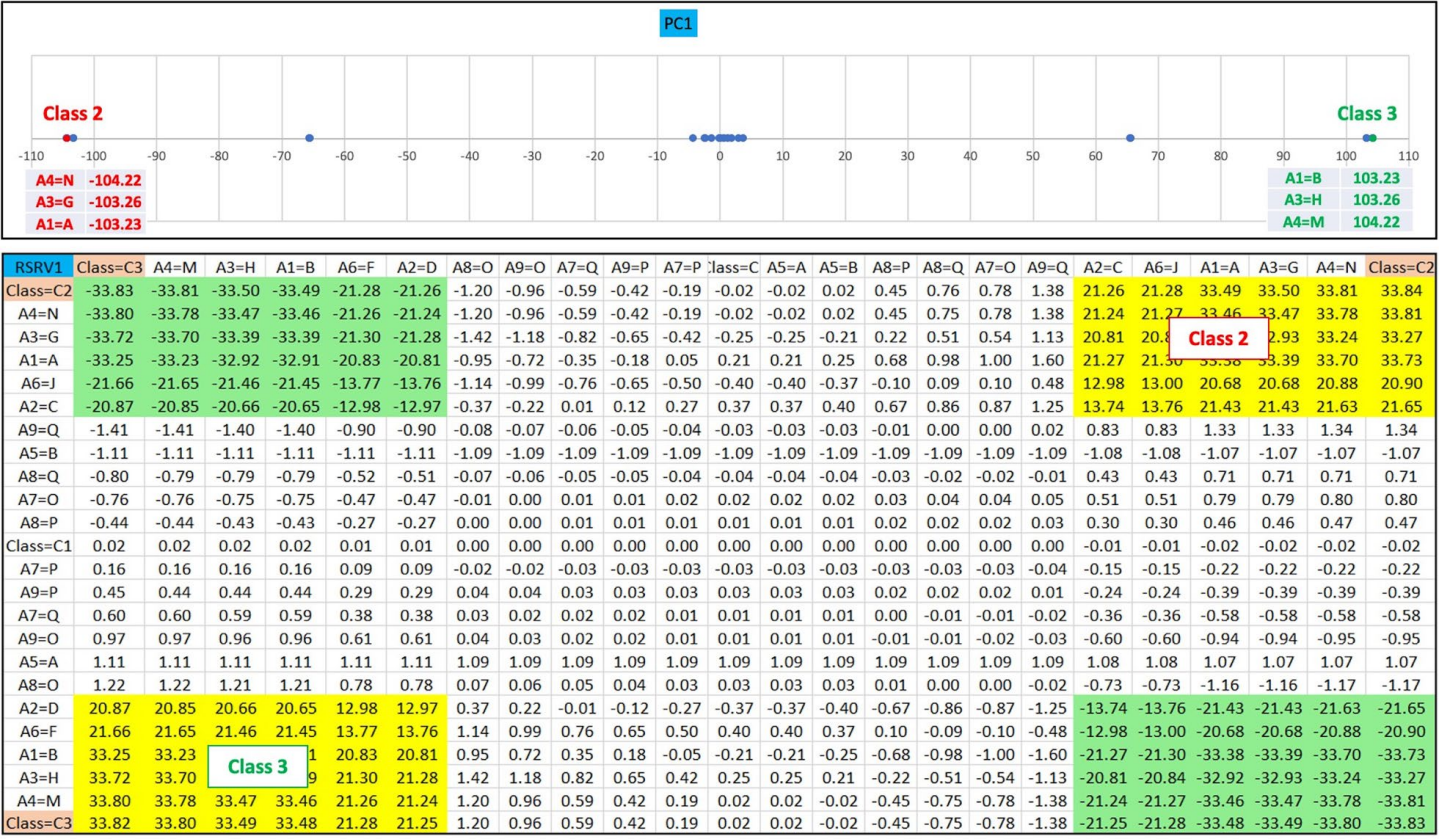
An illustration of DS* with two AV clusters in the first PC (PC1) using synthetic data. As displayed in PC1, two distinct clusters far from the centre represent two strongly AVA groups corresponding to class 2 and class 3 with large eigenvalue. The SR of their corresponding AVA among the AV pairs in each cluster are shown by the yellow shaded cells in its corresponding Re-projected SRV (RSRV). This indicates that the AVAs of class 2 and class 3 are disentangled and grouped in the first PC and its corresponding RSRV. The green shaded cells in RSRV denote AV pairs with negative statistical significance (i.e., very unlikely to occur)

2. **Acquisition of AVA Disentangled Spaces**: For AVA disentanglement, Principal Component Decomposition (PCD) is applied to decompose the SRV into *N* PCs, denoted as PC = { *PC*_1_, *PC*_2_, …*PC*_*k*_…*PC*_*N*_ }, where *PC*_*k*_ is a set of projections of the AV vectors obtained from SRV after the PCD, and *PC*_*k*_ = { ${PC}_k\left({A}_n^i\right) \mid n=1,2,\dots N,i=1,\dots {I}_n$ }. We then re-project the projections of the AV-vectors captured in each PC to a new SRV with the same basis vectors and call it a Reprojected SRV (RSRV) corresponding to that PC. We then refer all the PCs and the their corresponding RSRVs = { *RSRV*_1_, *RSRV*_2_, …*RSRV*_*k*_, …*RSRV*_*N*_ } as the AVA disentangled spaces (DSs) where *RSRV*_*k*_ is the re-projected result on *PC*_*k*_ via *RSRV*_*k*_ = *SRV* ∙ *PC*_*k*_ ∙ *PC*_*k*_^*T*^. Similar to SRV, each RSRV is an *I* × *I* matrix, and each row of a RSRV corresponding to an AV represents an AV-vector whose coordinates are the SR of that AV associating with other AVs represented by the column vectors in the RSRV. The coordinates of these AV vectors in the RSRV represent the SRs of the AVAs captured in the PCs. We refer to a PC with its RSRV as a Disentangled Space (DS). Figure 2 shows a DS (PC and RSRV) obtained from the synthetic dataset.
3. **Identification of functional sub-group (AV-Cluster)**: Since the number of DSs is as large as the number of AVs, we then devise a DS screening algorithm to select a small subset from DSs (denoted by DS*) such that the maximum SR in its RSRV exceeds a statistical threshold (say 1.44 at confidence level of 85%). In the PC and RSRV of each DS*, often only one or two disjoint AV clusters are found. Each cluster may contain a few subgroups. Hence, the complexity of the PD process is greatly reduced. The criterion to form an AV cluster is that each AV in the cluster must be in a significant AVA with other AV in the cluster. In the RSRV (Fig. 2), the cells with yellow and green shade show the AV pairs with positive and negative statistical significance respectively.
4. **Pattern Discovery**: High-order patterns can be discovered through identifying pattern candidates through an AV cluster identification and growing process. Formally, we denote a high-order pattern as *P*_*j*_ which consists of a subset of AVs with size ≥ 2. We use the adjusted residual [2] derived from the frequency of co-occurrences of *P*_*j*_ used in the hypothesis test to assess whether *P*_*j*_ is a statistically significant pattern. In order to keep the discovered patterns non-redundant, we only accept delta-closed patterns [16, 17] in the pattern discovery process. There might be more than one pattern identified in the AV cluster. We treat the union of the AVs making up patterns in one AV cluster or in one functional sub-group as the summarized super pattern. All patterns discovered by cPDD are listed as the comprehensive patterns.
5. **Interpretation and Prediction**: The AVs in each AV cluster/subgroup making up a summarized pattern pertaining to a designated class/group. In all our experiments, due to AVA disentanglement, the summarized patterns contain no or very few “either-or AVs” within the pattern. Hence, the summarized pattern is more succinct and easier to interpret. The high-order patterns in the comprehensive set can provide all the detailed patterns for interpretation and linkage to individuals and groups. Since the number of candidate AVs are few in the output of cPDD, so the number of patterns discovered in each DS* is extremely small. This is significantly different from traditional PD. For class prediction when class labels are given, we can discover the disentangled patterns associating with class labels from the training data. In testing, we apply the discovered summarized patterns associated with each specific class to predict whether the entity for testing belongs to that class. Let (*P*_*j*_, *C*) represents a summarized pattern *P*_*j*_ associated with class label *C*, and *E*_*i*_ represent the entity needed to be predicted. Based on the mutual information in statistical information theory, we can use the weight of evidence [18, 19] of all the AVs in the summarized patterns to determine whether the class label for *E*_*i*_, *C*(*E*_*i*_), will have higher weight than predicting it as pertaining to other classes.

## Results and discussion

In this study, we conducted experiments both on the synthetic and the clinical dataset with imbalanced classes.

### Materials

#### Dataset 1: synthetic dataset

To show the capability of cPDD in interpreting an imbalance dataset, a synthetic experiment was designed and conducted. We generated stochastically a 2100 × 10 matrix with the first column as the class label and others as attributes with character values stochastically generated from a uniform distribution. This represents a random relational dataset with attributes independent to each other. We then embedded patterns of three different classes *C*_1_, *C*_2_, and *C*_3_ for the first 6 attributes. We use A1A, A2C, for example, to respectively represent character value A and C for Attribute A1 and A2. The patterns implanted in the data are summarized in Table 2. Note that A1A and A2C are entangled (overlapping) for *C*_1_ and *C*_2_; A3H and A4M are entangled in *C*_1_ and *C*_3_; A5B and A6J are entangled in *C*_2_ and *C*_3_. For the last three attributes, we put in randomly selected characters from {“O”, “P”, “Q”} and for the 10th attribute we randomly embedded characters used for the three classes. Moreover, this synthetic Dataset was implemented as one with imbalanced class distribution with 1000 entities pertaining to *C*_2_ and *C*_3_ each, and 100 entities pertaining to *C*_1_.

**Table 2:** Synthetic dataset with embedded entangled patterns

Classes	Attribute Values are Significant Associated with Class Label
C1	A1A, A2C, A3H, A4M/N, A5A, A6F
C2	A1A, A2C/D, A3G, A4N, A5B, A6J
C3	A1B, A2D, A3H, A4M, A5B, A6F/J

#### Dataset 2: thoracic dataset

**Fig. 3:**
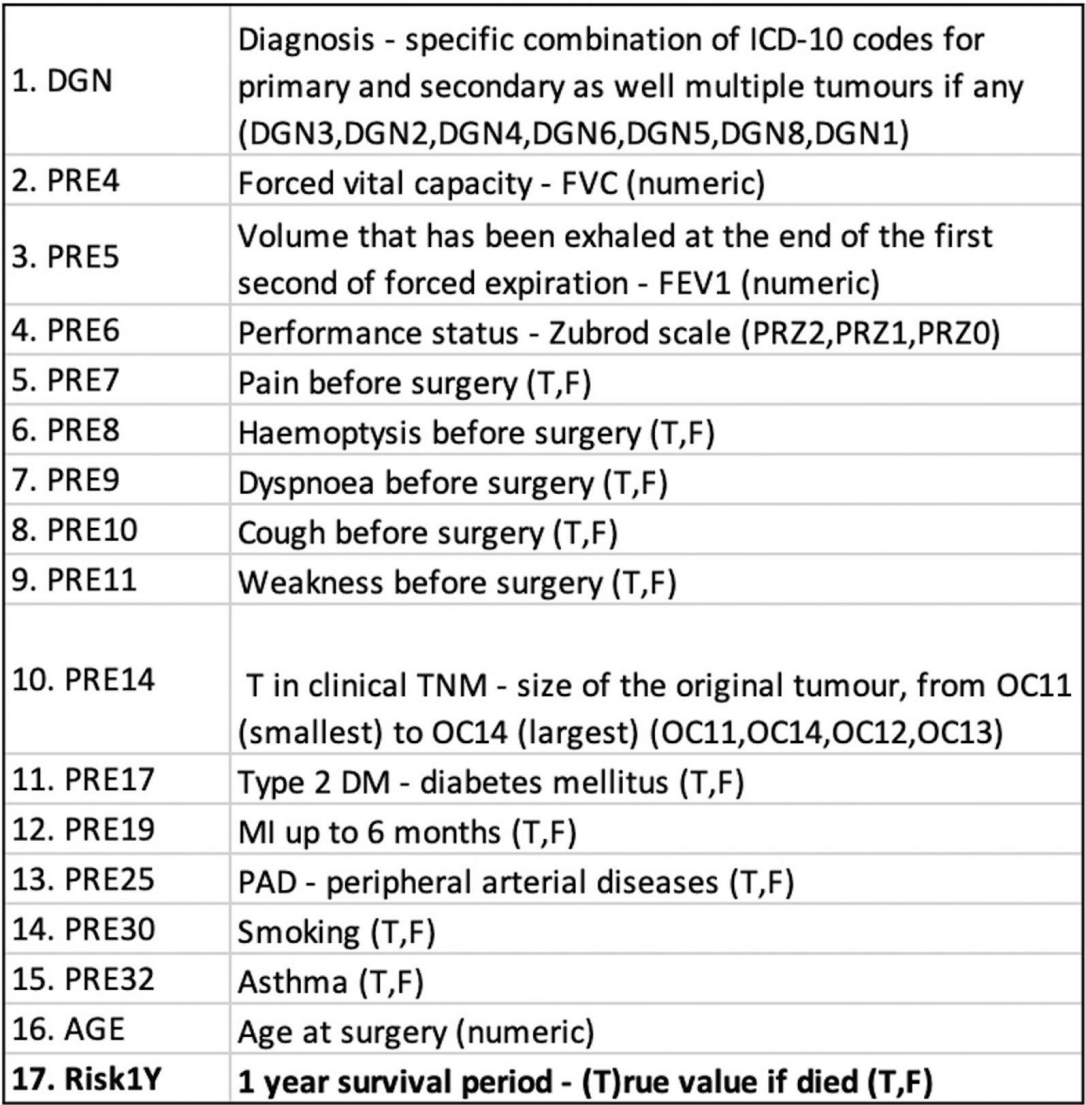
Attribute Description of Thoracic Dataset

The thoracic dataset describes the surgical risk originally collected at Wroclaw Thoracic Surgery Centre for patients who underwent major lung resections for primary lung cancer in the years 2007-2011 [20]. The attributes included are given in Fig. 3. This public dataset is provided after feature selection and elimination of missing values. It is composed of 470 samples with 16 pre-operative attributes after feature selection. The target attribute (class label) is Risk. Risk = T if the patient died. In this dataset, the class distribution is imbalanced with 70 cases being Risk = T and 400 cases being Risk = F. To simulate the target scenario without requiring much tweaking, the numeric attributes PRE4, PRE5 and age were removed.

### Analysis I – discovery and display of explicit patterns for explanation

**Fig. 4:**
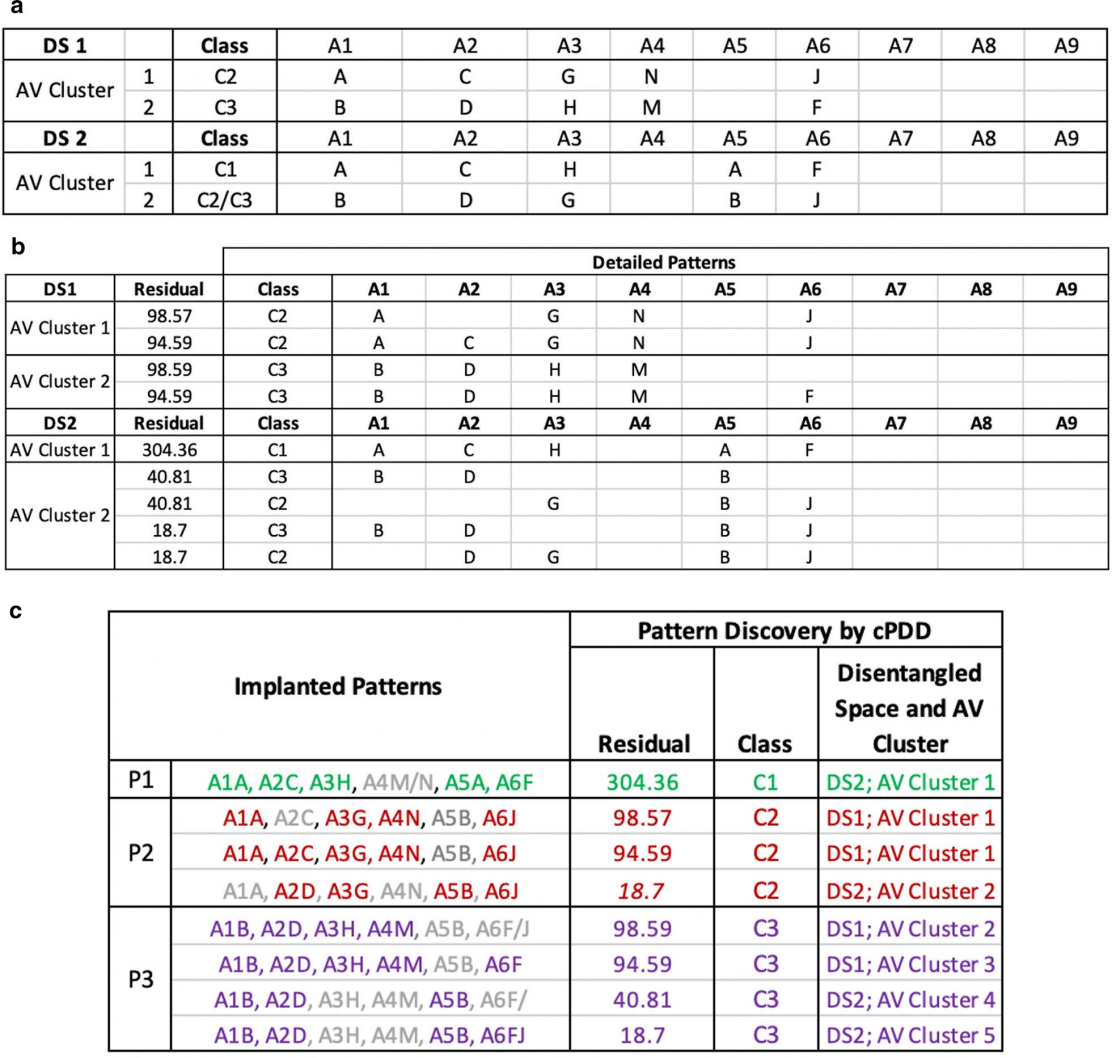
cPDD Pattern Discovery Result from Synthetic Dataset. **a** In the First and the Second DS* (DS 1 and DS 2) two AV-clusters were discovered. **b** Detailed Patterns associated with different classes were discovered from the above two AV-Clusters. **c** Implanted patterns showing close correspondence to the patterns obtained in cPDD’s output in (**a**) and (**b**)

**Fig. 5:**
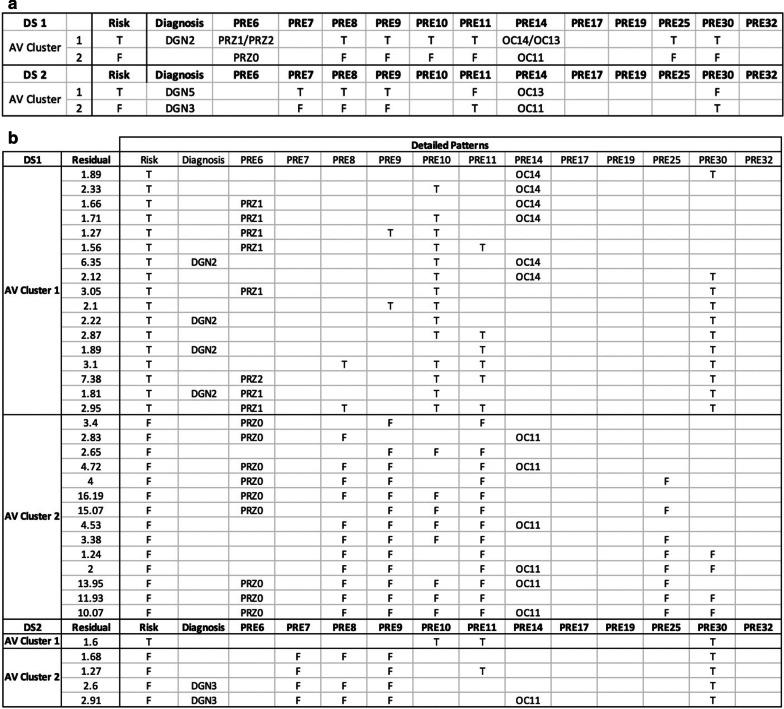
Pattern Discovery Result of Thoracic Dataset using cPDD **a** In both First and the Second Disentangled Space, two AV Clusters corresponding to Risk = T and RISK=F were discovered **b** Detailed Patterns discovered from the above two AV Clusters. Note that no AV of attributes PRE17, PRE19 and PRE32 are associated with any significant patterns. They were dismissed autonomously without undergoing feature engineering

**Fig. 6:**
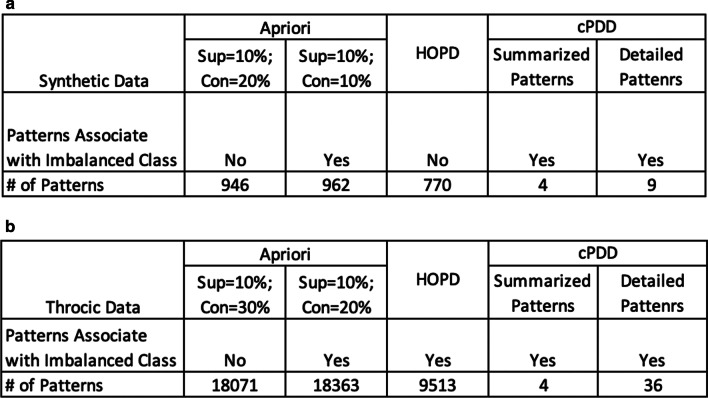
**a** Comparison of Pattern Discovery Result on Synthetic Dataset **b** Comparison of Pattern Discovery Result on Thoracic Dataset. The number of patterns discovered by cPDD is drastically reduced to enable succinct interpretation and the construction of a useful knowledge base

In Analysis I, we compared the discovered patterns obtained in cPDD, Apriori [21] (a typical frequent pattern mining method) and a high-order pattern discovery method for discrete-value data (HOPD) [6] which was our early work closely resembling the PD reported in [9, 10]. Figures 4 and 5 show the pattern discovery result of cPDD on the Synthetic and Thoracic data respectively. Figure 6 presents the comparison results of all these three methods.

As shown in Fig. 4, a small set of AV-Clusters was discovered from the synthetic dataset. Figure 4a displays the union of all the comprehensive patterns (Fig. 4b) and can be considered as the summarized pattern. The summarized pattern in each subgroup of a DS* consists the union of all the detailed patterns discovered in that subgroup of the DS*. While the summarized pattern gives a high-level interpretation of the AVs with significant AVAs in a subgroup, the detailed patterns encompass comprehensively all the significant patterns discovered in that subgroup with details and statistical support. In the like manner, the summarized patterns discovered form the Thoracic dataset are given in Fig. 5a and some samples of comprehensive set of patterns that are associated with class labels are displayed in Fig. 5b.

Figure 6 displayed the PD results obtained from cPDD and Apriori with two different fine-tuned sets of support and confidence on the synthetic dataset (Fig. 6a) and the Thoracic dataset (Fig. 6b). While cPDD discovers 4 summarized patterns and 9 detailed patterns from the synthetic dataset, the number of patterns discovered by Apriori and HOPD are overwhelming. For the thoracic dataset, similar comparative results for are shown in Fig. 6.

Furthermore, when comparing the implanted patterns with the patterns cPDD discovered (Fig. 4c), cPDD reveals all patterns with correct class labels in disentangled spaces except one in P2 as it has (A2D, A3G, A5B, A6J). Although this pattern is the same as the implanted patterns, yet it shares the sub-pattern (A2D, A5B, A6J) of P3 in C3 indicating the entanglement in the original data. Figure 4c shows high SR for the implanted patterns assigned with the correct classes and low SR for the entangled cases. For both Apriori or HOPD, they discovered a large number of patterns where most of them are redundant and overlapping. While some of them are associated with class labels, others are with the noise columns A7, A8 and A9 as well.

In addition, from the pattern discovery result on the Thoracic dataset, we observed similar phenomena if we replace Figs. 4 and 6a with Figs. 5 and 6b respectively. Figure 5a gives four AV-Clusters, two in each AVA disentangled Space (DS1 and DS2). Each AV-cluster contains the interpretable union of all the patterns discovered in different subgroups (Fig. 5a). A subset of the detailed patterns forming each union pattern is displayed in Fig. 5b.

Figure 6b shows the comparison results for the Thoracic data. First, the number of patterns obtained by Apriori and HOPD are both large. And it is difficult to interpret the pattern outcomes relevant to the problem when the number of patterns is large with considerable redundant and overlapping patterns. Second, Apriori outputs the patterns from datasets only if the class labels are given. HOPD can output all the patterns discovered among the growing set of the candidate patterns without knowing class labels, but the number of high order patterns produced are overwhelming. For a dataset **R** with *m* attributes, there are an exponential number of AV combinations being considered as pattern candidates. So, the number of patterns outputted by HOPD is huge. It is surprising to note that the highest order of patterns discovered for Thoracic Dataset by cPDD is 8 (Fig. 5b), yet the number of comprehensive patterns discovered is only 36 (Fig. 6b). This is really beyond what humans can grasp.

When we examined whether other algorithms could discover the patterns associated with the minority class, we found that the results of Apriori depend on the set value of the threshold, support, and confidence. When the threshold is low, more patterns are discovered which may cover those in the minority class, but the number of patterns is huge (Fig. 6b). When the set threshold was set high, patterns in the rare class were not discovered. As for HOPD, it discovered a large number of patterns that contain those of the rare classes. However, cPDD discovered a much smaller number of summarized and detailed patterns succinctly, including those from the rare class.

In summary, this experimental result shows that cPDD is able to discover fewer patterns with specific association to the classes in support of easy and feasible interpretation. Furthermore, even with few patterns, it is able to represent succinct, comprehensive (as exemplified in the synthetic case) and statistical/functional characteristics of all classes given, even when the class distribution is imbalanced. With the capability to render direct interpretation of a small, succinct and reliable set of patterns discovered from distinct sources without the reliance of explicit a priori knowledge and a posteriori processing, cPDD is a novel approach of Explainable AI (XAI) [22, 23] quite different from the existing model-based ML approach.

### Analysis II – prediction on imbalanced dataset

In Analysis II, we focus on the prediction of diagnostic outcomes of the Thoracic dataset with imbalance class distribution. We first report the testing results on the original thoracic dataset, then we cover the extended experiment results with sampling data.

#### Comparison result on original data

For the imbalanced class problem, since the correct prediction of the majority classes will overwhelm that of the minority classes, the prediction performance should not be evaluated based on the average accuracy [24]. Hence, in this study, the prediction results are evaluated by the F1-Score [25] calculated by Precision and Recall (or called sensitivity and specificity), Geometric mean of Precision and Recall (G-mean) [1] respectively for predicting the minority target.

The F1-Score for the minority class *C*_*m*_, denoted as *F*1(*C*_*m*_), can be calculated from the *Precision* (*C*_*m*_) and the *Recall* (*C*_*m*_) by Eq. (2).

2 $$F1\left({C}_m\right)=\frac{2\ast Recall\ \left({C}_m\right)\ast Precision\left({C}_m\right)}{Recall\ \left({C}_m\right)+ Precision\left({C}_m\right)}$$

where $Precision\ \left({C}_m\right)=\frac{TP}{\left( TP+ FP\right)}$, $Recall\ \left({C}_m\right)=\frac{TP}{\left( TP+ FN\right)}$, *TP* represents True Positive; *FP* represents False Positive, and *FN* represents False Negative when considering the minority class label as the target. Thus, according to the definition, F1-score = 0 if the number of true positive *TP* = 0.

The G-mean for the minority class, denoted as G-mean(*C*_*m*_), is calculated from the *Precision* (*C*_*m*_) and the *Recall* (*C*_*m*_) by Eq. (3).

3 $$G- mean\left({C}_m\right)=\sqrt{Recall\ \left({C}_m\right)\ast Precision\left({C}_m\right)}$$

We do not show the comparison result of the Precision or Recall of minority class since for the imbalanced data problem, if all the cases are detected as majority class target or minority class target, they may have extremely high precision or recall. Hence, the average F1-score or G-mean calculated by both Precision and Recall are obtained from 20-time-10-fold cross-validation used for performance evaluation.

Besides, considering both majority and minority decision in the comparison result, we list the average accuracy to show how misleading these results could be as they are unreliable measures for classification performance evaluation for the case with imbalanced classes. Compared to Accuracy and F1 score, MCC renders a more reliable statistical rate which produces a high score only if the prediction obtained good results in all of the four confusion matrix categories (TF, FN, TN and FP) [26]. The value of MCC is from − 1 to + 1, where + 1 represents a perfect prediction, 0 an average random prediction and − 1 an inverse prediction. Hence, wwe also list the Matthews correlation coefficient (MCC) [26] (Eq. (4)) and Balanced Accuracy (Balance Acc.) [27] to show the accuracy after the balanced results are obtained.

4 $${\displaystyle \begin{array}{c} MCC=\frac{\frac{TP}{N}-S\ast P}{\sqrt{PS\left(1-S\right)\left(1-P\right)}}\\ {}\mathrm{where}\ N= TN+ TP+ FN+ FP,S=\frac{TP+ FN}{N},P=\frac{TP+ FP}{N}\end{array}}$$

**Fig. 7:**
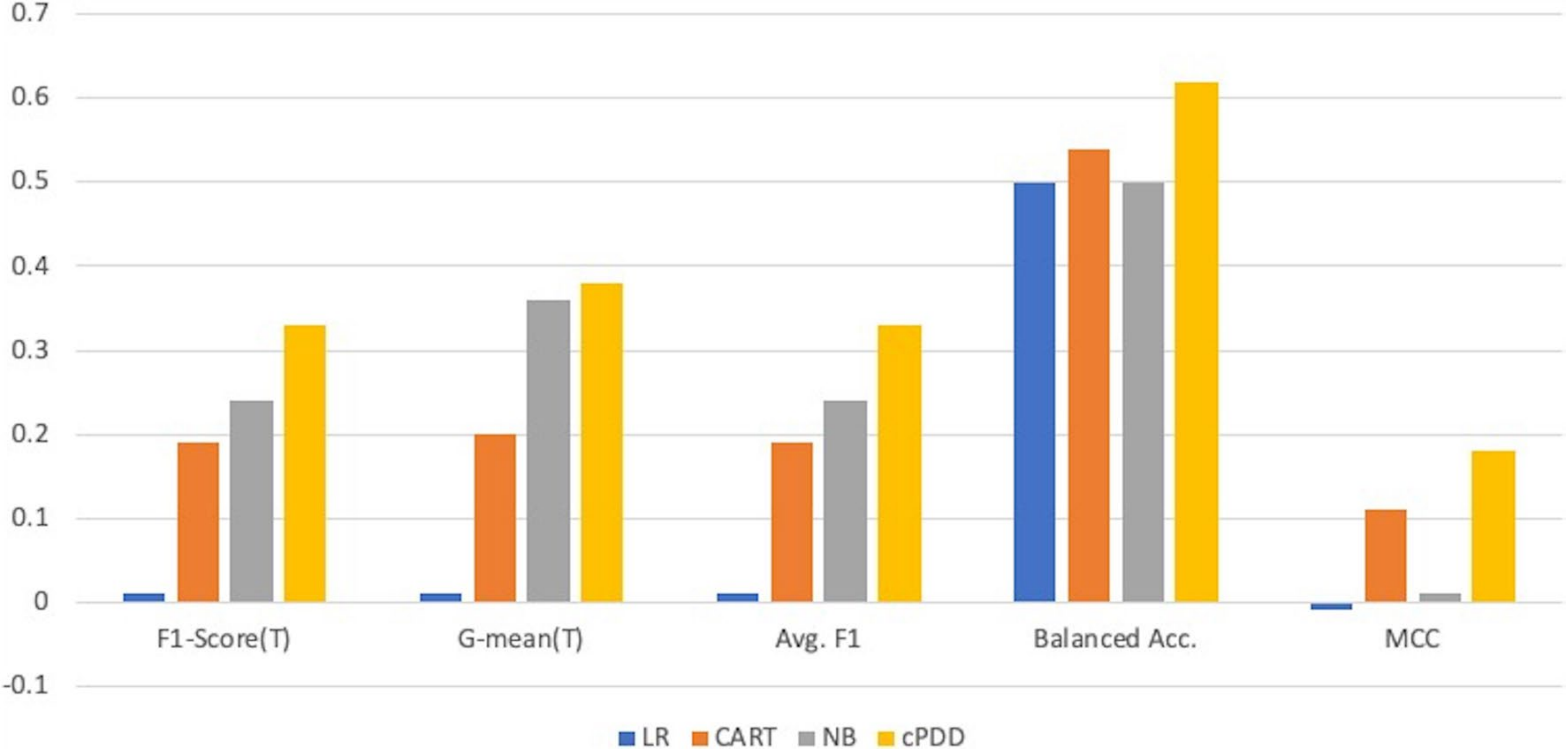
Comparison Result from 20-time-10-fold cross-validation on Thoracic Dataset

In this study, cPDD was compared with Logistic Regression (LR), Naïve Bayes (NB), and Decision Tree (CART). All of the above algorithms were implemented under default parameters using the Python machine learning package, scikit-learn 0.23.2 [28]. The comparison results are given in Table 3 and Fig. 7. All results are listed as *mean ± variance*.

**Table 3:** Comparison result from 20-time-10-fold cross-validation using different classification algorithm

Original Thoracic Data	LR	CART	NB	cPDD
F1-Score(T)	0.01 ± 0.00	0.19 ± 0.03	0.24 ± 0.01	0.33 ± 0.01
G-mean(T)	0.01 ± 0.01	0.20 ± 0.03	0.36 ± 0.01	0.38 ± 0.01
Avg. F1	0.01 ± 0.01	0.19 ± 0.03	0.24 ± 0.01	0.33 ± 0.01
Avg. Acc	0.84 ± 0.84	0.82 ± 0.01	0.15 ± 0.00	0.59 ± 0.01
Balanced Acc.	0.50 ± 0.00	0.54 ± 0.01	0.50 ± 0.00	0.62 ± 0.01
MCC	−0.01 ± 0.00	0.11 ± 0.03	0.01 ± 0.00	0.18 ± 0.01

*F1-Score(T)* Average F1-Score on Risk = T, *G-means(T)* Average G-mean on Risk = T, *Avg. F1* Average F1-Score for both classes (Risk = T and Risk = F), *Avg. Acc* Average of prediction accuracy on the whole dataset

In Table 3, LR shows poor prediction performance, resulting in 0.01 ± 0.00 on F1-Score and 0.01 ± 0.01 on G-mean. CART achieved a slightly better performance on F1-Score with 0.19 ± 0.03 and on G-mean with 0.20 ± 0.03 since the weighted samples were used for optimizing CART [28]. Naive Bayes was less influenced as the target proportion could be used as the prior information in training. Finally, we found that cPDD achieved the best performance on F1-Score with 0.33 ± 0.01 and G-means with 0.38 ± 0.01. In addition, for this set of imbalanced data, Balance Acc. would be better than regular accuracy [27]. Without sampling, both LR and NB shows the balanced accuracy as 0.5 which is same with randomly selection, even though NB shows better performance of classification for minority class.

Similarly, as Table 3 shows, CART obtained a slightly higher value on Balanced Acc and MCC. However, cPDD obtained the highest results for both Balanced Acc. with 0.62 + =0.01 and MCC with 0.18 + =0.01, as well as all the other scores except for Avg. Acc. which is not that meaningful for the imbalance cases.

In summary, cPDD has achieved the best result no matter whether it is applying on the entire dataset or only on the minority classes. This indicates that cPDD is more robust and reliable.

#### Comparison result on sampling data

To reduce the inaccuracy of such kind of biased classification, researchers usually use undersampling and oversampling methods to balance the samples of the training data [29]. Therefore, both random oversampling [30] and random undersampling [31] have been applied to the dataset before training and predicting.

##### Random oversampling

Random oversampling [30] duplicates records randomly from the minority class and adds them to the training dataset. However, oversampling may result in overfitting towards the minority class samples especially for higher over-sampling rates [29, 32]. To implement the random oversampling algorithm, we used the imbalanced-learn Python library [33].

In this experiment, we separated the training data and testing data first, and then applied random oversampling on the training data. The original training dataset would include 423 records with ~ 63 records taken from the minority class and ~ 360 from the majority class. After applying the above random oversampling method, the number of training records was increased to 720. To keep consistent with experiments on original data, we used the original 47 test samples (10% of the original entire set) with imbalanced classes as the test set. We then applied all classification methods for training and testing on this set of data. Since we have shown that the average accuracy should not be used as a reasonable approach for prediction evaluation for imbalanced dataset, we listed in Table 4 only the average F1-score and G-mean for minority class and the average F1-score for the entire dataset.

**Table 4:** Comparison result from 20-time-10-fold cross validation with different sampling strategies

	LR	CART	NB	cPDD
Over Sampling				
F1-Score(T)	0.31 ± 0.02	0.20 ± 0.02	0.26 ± 0.01	0.37 ± 0.01
G-mean(T)	0.34 ± 0.02	0.22 ± 0.22	0.36 ± 0.00	0.40 ± 0.01
Avg. F1	0.31 ± 0.02	0.20 ± 0.20	0.26 ± 0.01	0.59 ± 0.01
Balanced Acc.	0.61 ± 0.01	0.52 ± 0.01	0.50 ± 0.00	0.61 ± 0.01
MCC	0.17 ± 0.03	0.04 ± 0.02	0.01 ± 0.00	0.25 ± 0.01
Under Sampling				
F1-Score(T)	0.30 ± 0.01	0.27 ± 0.02	0.25 ± 0.01	0.34 ± 0.02
G-mean(T)	0.34 ± 0.01	0.30 ± 0.02	0.35 ± 0.02	0.41 ± 0.03
Avg. F1	0.30 ± 0.01	0.27 ± 0.02	0.25 ± 0.01	0.63 ± 0.02
Balanced Acc.	0.59 ± 0.01	0.57 ± 0.01	0.54 ± 0.01	0.61 ± 0.02
MCC	0.13 ± 0.02	0.11 ± 0.02	0.08 ± 0.02	0.20 ± 0.08

*F1-Score(T)* Average testing F1-Score on Risk = T, *G-means(T)* Average testing G-mean on Risk = T, *Avg. F1* Average testing F1-Score for both classes (Risk = T and Risk = F)

As the results show, cPDD still achieved superior prediction results, since it can handle imbalanced dataset effectively. The oversampling strategy did not change the result too much for cPDD as its F1-score and G-mean increased respectively to (0.37 ± 0.01) and (0.40 ± 0.01) from (0.33 ± 0.01) and (0.38 ± 0.01) in Table 3. Similarly, both CART and NB would be less influenced by imbalanced data as the results only slightly increased with F1-Score 0.20 ± 0.02 and G-mean 0.22 ± 0.22 for CART, and almost kept the same for NB with F1-Score 0.26 ± 0.01 and G-mean 0.36 ± 0.00. However, since Logistic Regression is not designed for imbalanced datasets, so after oversampling, the dataset became a balanced dataset, and the performance of LR improved considerably with F1-score (0.31 ± 0.02) and G-mean (0.34 ± 0.02) for the minority class.

##### Random undersampling

Similar to the random oversampling, random undersampling [31] randomly deletes records from the majority class. The process is repeated until the training dataset becomes a balanced dataset. The same Python library [33] was used for implementation. And the average F1-Score, G-mean for minority class and the average F1-Score, MCC and Balance Acc. for the entire testing data were used for evaluation.

In this experiment, after applying the random undersampling algorithm, the number of training dataset (423 records) was reduced to 126 since the size of majority class was reduced. Then the same classification methods were applied to the same testing dataset.

As Table 4 shows, cPDD still achieved superior prediction results with F1-score (0.34 ± 0.02) and G-mean (0.41 ± 0.02). The results were also improved for CART with F1-Score in 0.27 ± 0.02 and G-mean in 0.30 ± 0.02 whereas the results of NB were least influenced by undersampling with F1-Score in 0.25 ± 0.01 and G-mean in 0.31 ± 0.02 while those of Logistic Regression improved with F1-score (0.30 ± 0.01) and G-mean (0.34 ± 0.01) for the minority class.

**Fig. 8:**
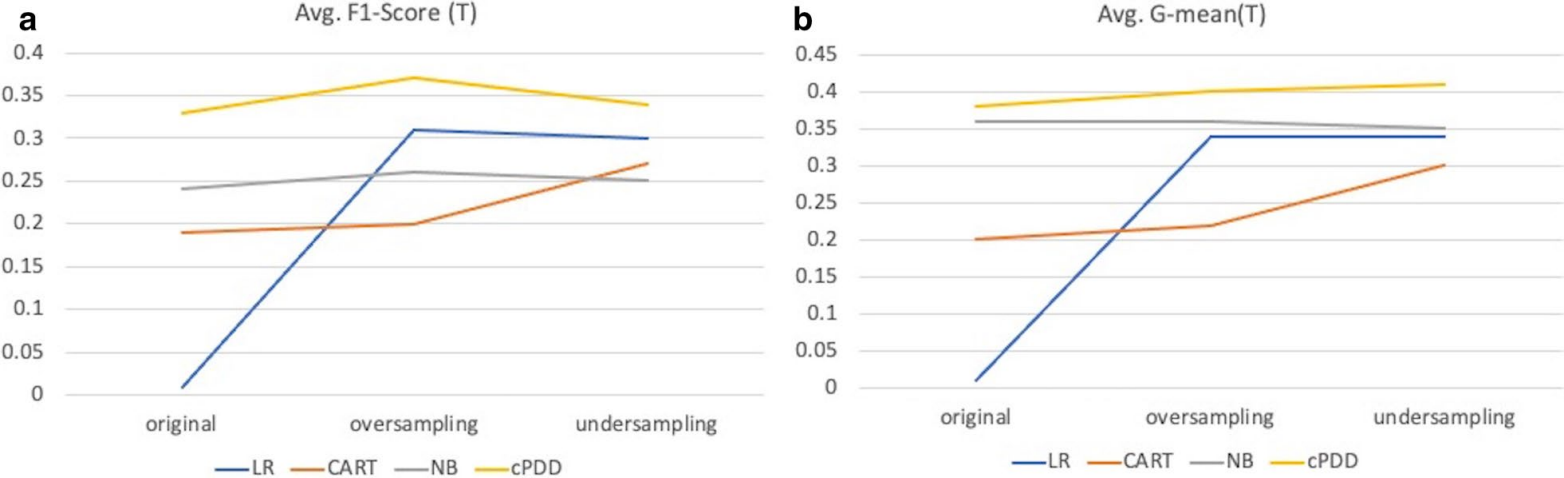
Comparison Results from 20-time-10-fold cross validation on Risk = T with different sampling strategies. **a** Comparison of average testing F1-Score Using Various Classification Algorithms **b** Comparison of average testing G-mean Using Various Classification Algorithms

Besides, the comparison plots of F1-Score and G-mean are shown in Fig. 8. As the Fig. 8 shows, both cPDD and NB were less influenced by imbalanced data because both of them are “probabilistic classifiers” using statistical theory. cPDD could achieve better performance because it can discover even the hidden patterns. Logistic Regression cannot handle imbalanced data, so after samplings, the performance of LR improved considerably. Comparing between different sampling strategies, we found that CART could achieve better results when undersampling strategy was applied, and LR could obtain better results when oversampling strategy was applied.

In summary, without sampling, cPDD showed robust prediction performance in comparison with all other methods. And for all sampling strategies, cPDD still performed best. Thus, no matter whether the data is balanced or imbalanced, cPDD can handle it robustly and steadily.

## Conclusion

As a pattern discovery method on imbalanced synthetic and Thoracic data, cPDD renders a much smaller succinct set of explicit class-associated patterns for better interpretation, and superior prediction since it uses disentangled patterns which are more specific and distinct to the classes. The results it obtains are statistically robust with comprehensive coverage of succinct, concise, precise, displayable and less redundant representations for experts’ interpretation. cPDD also overcomes the limitations of lack of transparency [11] as well as the problem of imbalanced class [2, 3, 11, 34]. As a clinical data analysis tool on relational data, it has a significant advantage over the ‘black box’ ML algorithms since its output of is both transparent and interpretable, the two major challenges of interpretability and applicability [22] confronting ML on relational data today. The experimental result on synthetic and clinical data with high imbalanced class ratios shows that cPDD does have a superior prediction and interpretability performance for minority targets. cPDD brings explainable AI to clinical experts to enhance their insight and understanding with statistical and rational accountability. Hence, it will have great potential to enhance ML and Explainable AI [22, 23].

In our future work, cPDD will be developed to apply to unstructured data (e.g., text and sequences) [7, 35] by extracting patterns directly from them as shown in our early work [36]. Moreover, for performance improvement, parallel computing strategy will be introduced to handle bigger data and further speed up the computational time.

## Data Availability

The thoracic dataset is available at from the University of California Irvine Machine Learning Repository: https://archive.ics.uci.edu/ml/datasets/Thoracic+Surgery+Data.

## Abbreviations

AV: Attribute value
AVA: Attribute Value Association
AV Cluster: Attribute Value Cluster
SR: Adjusted Statistical Residual for an AV pair
SRV: AVA Adjusted Statistical Residual Vector Space
PCD: Principal component decomposition
RSRV: Re-projected SRV
DS: Disentangled Space
DS*: Selected Disentangled Space, the selected set
